# Schizophrenia Shows Disrupted Links between Brain Volume and Dynamic Functional Connectivity

**DOI:** 10.3389/fnins.2017.00624

**Published:** 2017-11-07

**Authors:** Anees Abrol, Barnaly Rashid, Srinivas Rachakonda, Eswar Damaraju, Vince D. Calhoun

**Affiliations:** ^1^The Mind Research Network, Albuquerque, NM, United States; ^2^Department of Electrical and Computer Engineering, University of New Mexico, Albuquerque, NM, United States

**Keywords:** multimodal fusion, structure-function relationship, schizophrenia, gray matter, dynamic functional connectivity, mCCA, joint ICA

## Abstract

Studies featuring multimodal neuroimaging data fusion for understanding brain function and structure, or disease characterization, leverage the partial information available in each of the modalities to reveal data variations not exhibited through the independent analyses. Similar to other complex syndromes, the characteristic brain abnormalities in schizophrenia may be better understood with the help of the additional information conveyed by leveraging an advanced modeling method involving multiple modalities. In this study, we propose a novel framework to fuse feature spaces corresponding to functional magnetic resonance imaging (functional) and gray matter (structural) data from 151 schizophrenia patients and 163 healthy controls. In particular, the features for the functional and structural modalities include dynamic (i.e., time-varying) functional network connectivity (dFNC) maps and the intensities of the gray matter (GM) maps, respectively. The dFNC maps are estimated from group independent component analysis (ICA) network time-courses by first computing windowed functional correlations using a sliding window approach, and then estimating subject specific states from this windowed data using temporal ICA followed by spatio-temporal regression. For each subject, the functional data features are horizontally concatenated with the corresponding GM features to form a combined feature space that is subsequently decomposed through a symmetric multimodal fusion approach involving a combination of multiset canonical correlation analysis (mCCA) and joint ICA (jICA). Our novel combined analyses successfully linked changes in the two modalities and revealed significantly disrupted links between GM volumes and time-varying functional connectivity in schizophrenia. Consistent with prior research, we found significant group differences in GM comprising regions in the superior parietal lobule, precuneus, postcentral gyrus, medial/superior frontal gyrus, superior/middle temporal gyrus, insula and fusiform gyrus, and several significant aberrations in the inter-regional functional connectivity strength as well. Importantly, structural and dFNC measures have independently shown changes associated with schizophrenia, and in this work we begin the process of evaluating the links between the two, which could shed light on the illness beyond what we can learn from a single imaging modality. In future work, we plan to evaluate replication of the inferred structure-function relationships in independent partitions of larger multi-modal schizophrenia datasets.

## Introduction

Structural neuroimaging modalities evaluate anatomical brain structure and tissue type (e.g., structural MRI or sMRI) or brain tissue microstructure (e.g., diffusion MRI or dMRI), whereas functional neuroimaging modalities indirectly estimate brain function/activity through respective characteristic “source signals” or “indicators” of the underlying neuronal (e.g., electroencephalography or EEG, magnetoencephalography or MEG), metabolic (e.g., positron emission tomography or PET) or hemodynamic (e.g., functional MRI or fMRI) activity. As the modalities are acquired at different spatial and temporal scales, the spatiotemporal precisions can be enhanced immensely, for example, by combining a modality with superior spatial resolution with another modality with superior temporal resolution. Furthermore, if the modalities are generically thought of as producing filtered sights of brain's organization or activity, working with multiple modalities would enable complementary sights into brain structure and/or function, thus intrinsically accomplishing a more comprehensive view (Calhoun et al., 2006; Calhoun and Adali, 2009; Schultz et al., 2012; Sui et al., 2012b; Uludag and Roebroeck, 2014; Calhoun and Sui, 2016). Given the above benefits, multimodal neuroimaging data acquisition and analysis has become much more widely utilized in recent years.

In a multimodal study, data corresponding to the different modalities might be acquired separately or simultaneously depending on the research question being addressed. Generically, separate data acquisition results in marginally higher signal to noise ratio (SNR) and lesser artifacts. However, simultaneous data acquisition is essential in studies where the objective is to study time-dependent responses to events, and inter-modality correlates (Uludag and Roebroeck, 2014). As an example, it would be important to simultaneously acquire EEG and fMRI data if the study goal is to identify potential correlates of time-varying functional connectivity measures in fMRI data to the EEG data. In this case, the acquired modalities could be analyzed through separate or collective pipelines using a variety of univariate or multivariate algorithm through a model-based or data-driven approach (Calhoun and Sui, 2016). Previous multimodal work has typically analyzed data from different modalities separately and correlated the independent results from the unimodal analyses, or used one of the modalities to constrain models corresponding to the other modality. The above mentioned types of multimodal studies have proven to be very useful, but make minimal or limited use of the cross-modality (i.e., joint) information, a resource that is now being increasingly availed by use of “symmetric” data fusion approaches (Calhoun and Sui, 2016). “Feature-based” symmetric data fusion approaches inherently first estimate useful features from the different modalities independently and then evaluate relationships between these features. This practice leverages the partial information available in each of the modalities to reveal data variations not exhibited through the independent analyses. To date, there have been several interesting demonstrations of the potential of utilizing such cross-modality or joint information in understanding the human brain and its disorders, disease characterization or biomarker identification, and uncovering disrupted links in complex mental illness (see Calhoun and Sui, 2016 for a detailed review).

Notably, multimodal studies with advanced modeling methods assume greater significance in diagnosis of a complex syndrome, for example schizophrenia, where striking pathological and etiological heterogeneity has been observed. Several previous studies (Olesen et al., 2003; Bassett et al., 2008; Hagmann et al., 2008; Rykhlevskaia et al., 2008; Honey et al., 2009; van den Heuvel et al., 2009; Camara et al., 2010; Michael et al., 2010; Skudlarski et al., 2010; Yu et al., 2011; Segall et al., 2012; Alexander-Bloch et al., 2013) clearly suggest interactions between structural and functional connectivity. Thus, it is reasonable to hypothesize covariation between “feature spaces” i.e., distilled (or lower dimensional or second/higher order) measures of brain structure and function in each modality. Importantly, reducing or projecting the very high dimensional data to feature spaces facilitates removal of redundant data while promoting identification of inter-modality relationships in a simpler, lower-dimensional space. Hence, in symmetric fusion approaches, it is the lower dimensional feature spaces that are fused to extract joint information. Some examples of lower dimensional feature spaces include contrast maps, amplitude of low frequency fluctuation maps (ALFF), etc. for fMRI data, segmented gray or white matter maps for sMRI data, fractional anisotropy (FA) or mean diffusivity (MD) maps for diffusion tensor imaging (DTI) data, single-nucleotide polymorphism (SNP) or methylation data for genetic data, etc.

Feature space projection is carried on by using a wide range of model-driven or data-driven approaches as reviewed in Calhoun and Sui (2016). Model-driven approaches have their own benefits in the form of enabling specific hypothesis testing of inter-regional interaction (provided there is enough prior information available on the problem being studied). On the other hand, data-driven approaches, in general, require specification of lesser assumptions about the data upfront. This makes them (the data-driven approaches) more suitable for studying complex problems, for instance a complex syndrome, such as schizophrenia, wherein little reliable prior knowledge is available. Data-driven approaches typically explore use of a blind or semi-blind multivariate approach so as to reveal hidden structure of inter-relationships between two (or more) data feature spaces. The use of multivariate approaches enables estimation of multiple variables jointly, and has some additional advantages over the use of univariate approaches. Multivariate approaches are relatively easy to interpret due to co-varying nature of the variables (i.e., regions of interest) and warrant additional robustness to noise as measures from patterns are explored rather than measures from paired relationships (Calhoun and Sui, 2016). Recently used blind multivariate decomposition methods include, but are not limited to, joint independent component analysis (jICA) (Calhoun et al., 2006), multiset canonical correlation analysis (mCCA) (Correa et al., 2007), partial least squares (PLS) (Martinez-Montes et al., 2004; Chen et al., 2009), and linked ICA (Groves et al., 2011), while adaptive (semi-blind) approaches, such as coefficient constrained ICA (CC-ICA) (Sui et al., 2009), and parallel ICA (Liu et al., 2009) also exist.

The above discussed multivariate approaches are all based on linear mixture models, but differ considerably in the optimization strategies/priorities they evolve the data sources through as well as in their basic limitations. Additionally, combining multiple multivariate algorithms has also been recently suggested to allow flexibility in the estimations by reducing the limiting effects of the individual approaches (Sui et al., 2011) as discussed next. The joint sources estimated by the jICA (or the linked ICA) algorithm are optimally maximally independent but share a common mixing matrix, thus assuming a very strong correlation between the joint sources. Contrarily, the mCCA algorithm jointly maximizes the inter-subject covariations thus allowing for varying levels of connectivity strengths between the joint sources. In this method, each dataset is decomposed into a set of sources with corresponding mixing profiles, also termed as canonical variates (CVs), and their corresponding correlation values, also called canonical correlation coefficients (CCCs). Despite allowing for varying activation levels of the joint sources, there remains the possibility that the spatial maps of the emergent joint sources in mCCA are highly similar in some cases where, for example, the CCC estimates are not sufficiently distinct (Correa et al., 2010; Sui et al., 2012a, 2013). Sui et al. (2011) used the mCCA+jICA algorithm for fusing the fMRI contrast maps and DTI FA maps to investigate group differences in healthy controls, schizophrenia patients and bipolar patients. Interestingly, this study concluded increased group classification accuracy with this algorithm as compared to its constituent algorithms tested alone. The combined mCCA+jICA model basically uses mCCA in the first step (Sui et al., 2011, 2013) followed by joint ICA (jICA) in the second step. In the first step, the different feature spaces are first linked with flexible linkages, thus adding to the investigator's confidence to perform jICA with an objective of identifying both highly and weakly correlated joint sources in the second step. A recent paper has proposed a unifying framework to link together a wide variety of multimodal approaches including the ones mentioned above (Silva et al., 2016).

Studies leveraging the above mentioned multivariate approaches have revealed significant information on clinical aspects of schizophrenia as discussed in several recent reviews on multimodal fusion (Biessmann et al., 2011; Schultz et al., 2012; Sui et al., 2012a; Lahat et al., 2015; Calhoun and Sui, 2016). Simultaneous analysis of anatomical and functional connectivity in Skudlarski et al. (2010) suggested that fusion allowed identification of deficits in white matter anatomy, and complex alterations in functional connectivity. In another multivariate, multimodal analysis, Michael et al. (2010) fused structural and functional brain images to reveal decreased overall structure-function linkage in schizophrenia as compared to healthy controls both in a working memory and an auditory sensorimotor task. Camchong et al. (2011) revealed convergent findings in multiple modalities (DTI and fMRI) consistent with the disconnection hypothesis in medial frontal regions in subjects with schizophrenia. jICA was used in Sugranyes et al. (2012) to characterize linked functional and white-matter changes related to working memory dysfunction, and in Stephen et al. (2013) to identify structure-function relationships using MEG and DTI modalities. The latter study concluded impairments in a posterior visual network in schizophrenia, with reduced FA and MEG amplitude, and overall weaker cognitive performance. Furthermore, Xu et al. (2009b) used joint source based morphometry (joint SBM) to identify linked white and gray matter (GM) differences in regions comprising temporal-corpus callosum, occipital/frontal-inferior fronto-occipital fasciculus, parietal/frontal-thalamus, and frontal/parietal/temporal-superior longitudinal fasciculus. Using the mCCA multivariate algorithm, Sui et al. (2015) identified linked structural and functional deficits in distributed cortico-striato-thalamic circuits and their association with cognitive impairments as measured through the Measurement And Treatment Research to Improve Cognition in Schizophrenia (MATRICS) consensus cognitive battery (MCCB). Finally, several classification studies have made use of multivariate, multimodal approaches to demonstrate improved classification with use of multiple modalities as compared to the use of a single modality in classifying patients from controls (Yang et al., 2010; Ulaş et al., 2011; Nieuwenhuis et al., 2012; Sui et al., 2013; Peruzzo et al., 2015; Cabral et al., 2016; Cetin et al., 2016).

Recent work assessing dynamic (i.e., time-varying) functional network connectivity (dFNC) suggests availability of substantial information beyond time-averaged functional connectivity (FC) estimates in both resting state and task-based fMRI data (Hutchison et al., 2013a; Calhoun et al., 2014; Preti et al., 2016). The term “chronnectome” was recently introduced to describe a focus on identifying whole brain transient and recurring patterns in temporal coupling of the human brain (Calhoun et al., 2014). The temporal dynamics of the time-courses are often characterized by the sliding window correlation (SWC) method (Sakoglu et al., 2010; Allen et al., 2012) to estimate the windowed functional network connectivity (wFNC) data followed by a rigorous FC “state” estimation process from the wFNC data (Allen et al., 2012; Miller et al., 2016). These FC states have been proven to be stably present in the data and reoccurring over time in a highly structured manner in our recent evaluations of replicability of time-varying FC states and state summary measures (Abrol et al., 2016, 2017). Several studies have shown the emergent states to be functionally and behaviorally relevant by demonstrating direct links with signatures of consciousness (Hutchison et al., 2013b; Amico et al., 2014; Hudson et al., 2014; Barttfeld et al., 2015; Wang et al., 2016), tracking day-dreaming/mind-wandering (Kucyi and Davis, 2014; Kucyi, in press), sleep and awake states (Tagliazucchi and Laufs, 2014), ongoing cognitive function and performance (Craddock et al., 2012; Schaefer et al., 2014; Gonzalez-Castillo et al., 2015; Madhyastha et al., 2015; Shine et al., 2016a,b). Furthermore, evidence of potential electrophysiological signatures of dynamic blood-oxygen-level dependent (BOLD) FC also hints the fluctuations in the BOLD FC (as captured in the states) to be interesting i.e., having a neurophysiological origin (Tagliazucchi et al., 2012; Chang et al., 2013; Allen et al., 2017), although further confirmation is still needed. Besides, several studies have also used time-varying connectivity measures to characterize pathophysiology i.e., identification of disease states (Damaraju et al., 2014; Rashid et al., 2014; Yu et al., 2015; Du et al., 2016; Miller et al., 2016).

**Figure 1:**
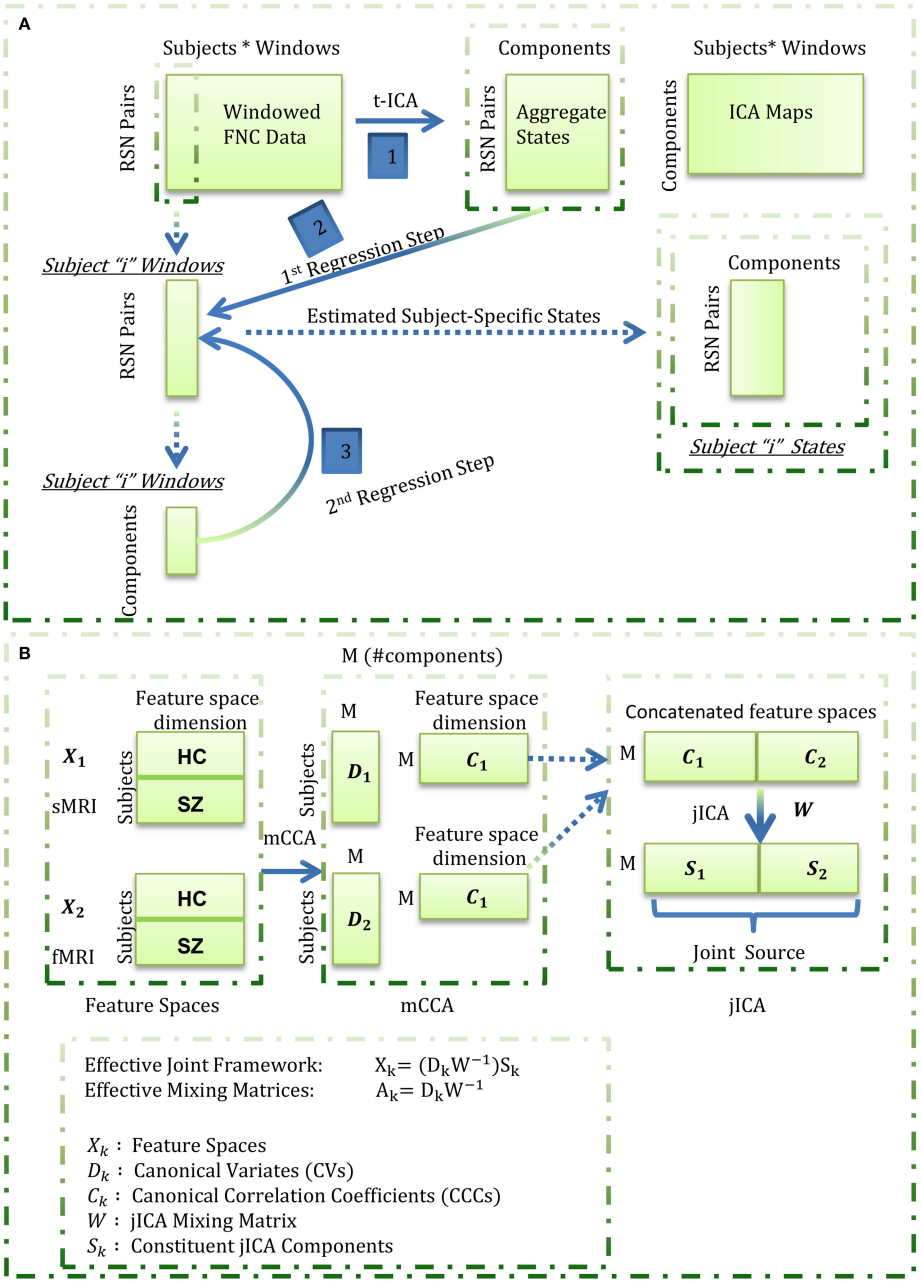
Estimation of the functional (fMRI) data feature space. Aggregate states were estimated by decomposing the windowed correlations by temporal ICA. Subject-specific states were next estimated through a spatio-temporal (dual) regression procedure wherein, for each subject, the aggregate states were regressed into the subject's windowed FNC data to estimate subject-specific component time-courses in the first regression step, and the estimated time-courses were regressed into the subject's windowed FNC to derive the subject-specific states in the second regression step; **(B)** Summary of the mCCA + jICA framework. For each subject, the functional data feature space as estimated in **(A)** was concatenated with the smoothed, modulated and warped gray matter maps (as the structural data feature space) and fused using the joint “mCCA+jICA” framework. This framework combines the mCCA and jICA algorithms to decompose the observed data into a linear combination of sources mixed through “effective” modality-specific mixing matrices as illustrated above.

In this work, we focus on feature based fusion analysis of brain structural (sMRI) and functional (fMRI) images hypothesizing correspondence between brain structure and function (or more specifically, correspondence between the feature spaces of the two studied modalities). We propose exploring where and how GM corresponds to time-varying functional connections will improve our understanding of both structural and functional connectivity. We estimate the feature space for the functional data as subject-specific states that are revealed from the wFNC data using a novel framework featuring temporal ICA and dual regression (Figure 1A). More specifically, aggregate states are estimated by decomposing the wFC data using temporal ICA in the first step, followed by a dual regression analysis to estimate the subject-specific states in the second step. This derived feature space from fMRI data, referred to as “functional data feature space” hereon, is simultaneously analyzed with the corresponding “structural data feature space,” i.e., GM maps from sMRI data, using the mCCA+jICA data fusion algorithm.

The fusion analysis in this work could be explained in a four-stage process (Figure 1B). In the first stage, mCCA reveals links between the modalities by maximizing the correlations between their mixing matrices i.e., CVs. In the second step, the associated maps to the CVs, i.e., the CCCs, are concatenated and decomposed using jICA to estimate the joint sources. In the third step, the (effective) modality-specific mixing matrices are estimated for the combined framework and analyzed for group differences for each joint source. In the last step, we focus on qualitative analysis of the subset of joint sources that feature linked, highly correlated and significant group difference showing structural and functional component maps from the jICA decomposition. Our exploratory investigation on data from 151 schizophrenia patients and 163 healthy controls shows general correspondence between GM and time-varying FC and also reveals few missing links in schizophrenia.

## Materials and methods

### Data acquisition and pre-processing

This study worked with T1-weighted structural and T2^*^-weighted resting state (subjects instructed to keep eyes closed but stay awake) functional images from 151 schizophrenia patients (114 males, 37 females; average age 37.8), and age and gender matched 163 healthy controls (117 males, 46 females; average age 36.9). This data were collected at seven different sites across the United States as a part of the function biomedical informatics research network (fBIRN) data repository (Keator et al., 2016). Informed consent was received from the participants as per institutional guidelines practiced at the seven collection sites. Six sites used the 3T Siemens Tim Trio System while one site used the 3T General Electric Discovery MR750 scanner. A total number of 162 volumes of standard gradient echo planar imaging (EPI) BOLD fMRI data were captured with a repetition time (TR) of 2 s, echo time (TE) of 30 s, field of view (FOV) of 220 × 220 mm^2^ (64 × 64 matrix), flip angle (FA) of 77° and 32 sequential ascending axial slices of 4 mm thickness and 1 mm skip.

The sMRI data were spatial normalized, bias corrected, and segmented using the SPM unified segmentation model in an automated analysis pipeline developed at the Mind Research Network (Bockholt et al., 2010) to obtain the smoothed (using a full width half maximum Gaussian kernel of 10 mm), modulated and warped GM images for all subjects. The GM maps were then used as the input feature space to the fusion algorithm with an objective of estimating the patterns of brain structure that exhibit co-variations across subjects. The fMRI data were pre-processed using the SPM, AFNI and GIFT toolboxes as well as custom code written in Matlab in a similar fashion as Damaraju et al. (2014). Briefly, rigid body motion correction was performed using the INRIAlign SPM toolbox for subject head motion correction. This was followed by slice-timing correction step in order to account for any timing differences in scan acquisition following which the data were de-spiked using AFNI's “3dDespike” algorithm so as to reduce the impact of outliers. Next, the data were warped to the Montreal Neurological Institute (MNI) template and resampled to 3 mm cubic isotropic voxels. Since the fBIRN data came from multiple sites, the site or scanner variability needed to be smoothed equivalently. This was done using AFNI's “BlurToFWHM” algorithm, an approach that has been shown to decrease scanner-specific variability in smoothness and provide “smoothness equivalence” to the multi-site data (Friedman et al., 2008). Finally, the voxel time-courses were variance normalized before running the group ICA.

### Functional data feature space

#### Group ICA, resting state network (RSN) selection and post-processing

The pre-processed fMRI data were decomposed using spatial group ICA to reveal spatially independent components each with a unique time-course profile (Calhoun et al., 2001; Calhoun and Adali, 2012). The pre-processed datasets were first reduced to 130 principal components in the time-point dimension at the subject level. Using a (relatively) higher number of principal components at the subject level has been shown to stabilize back-reconstruction and retain maximum variance in the data, and if this is the case the specific number does not substantially impact the results (Erhardt et al., 2011). Accordingly, the entire dataset was transformed into 130 principal components using standard principal component analysis (PCA) at the subject level in the first data reduction step of the group ICA analysis (similar to Damaraju et al. (2014) on the same fBIRN phase 3 dataset). Using a relatively high number of principal components in this step retained a very high percentage of subject level variance (>99.99%). In the second data reduction step, the PCA reduced subject data were then concatenated along the time dimension and further reduced to 100 components by implementing group level PCA. A higher model order for group ICA was chosen to enable a more refined (i.e., finer) RSN parcellation (Kiviniemi et al., 2009; Abou-Elseoud et al., 2010), thus allowing evaluation of sub-nodes within network domains (Allen et al., 2012; Sockeel et al., 2016; Abrol et al., 2017; Li et al., 2017; Fu et al., in press). The reliability of the estimated independent components from this step was evaluated using ICASSO (Himberg et al., 2004), and it was found that the estimates exhibited tight clustering and converged consistently amongst several (twenty) runs. Finally, subject-specific component spatial maps (SMs) and time-courses (TCs) were estimated using the GICA back-reconstruction methods as implemented in the GIFT toolbox (Erhardt et al., 2011).

**Figure 2:**
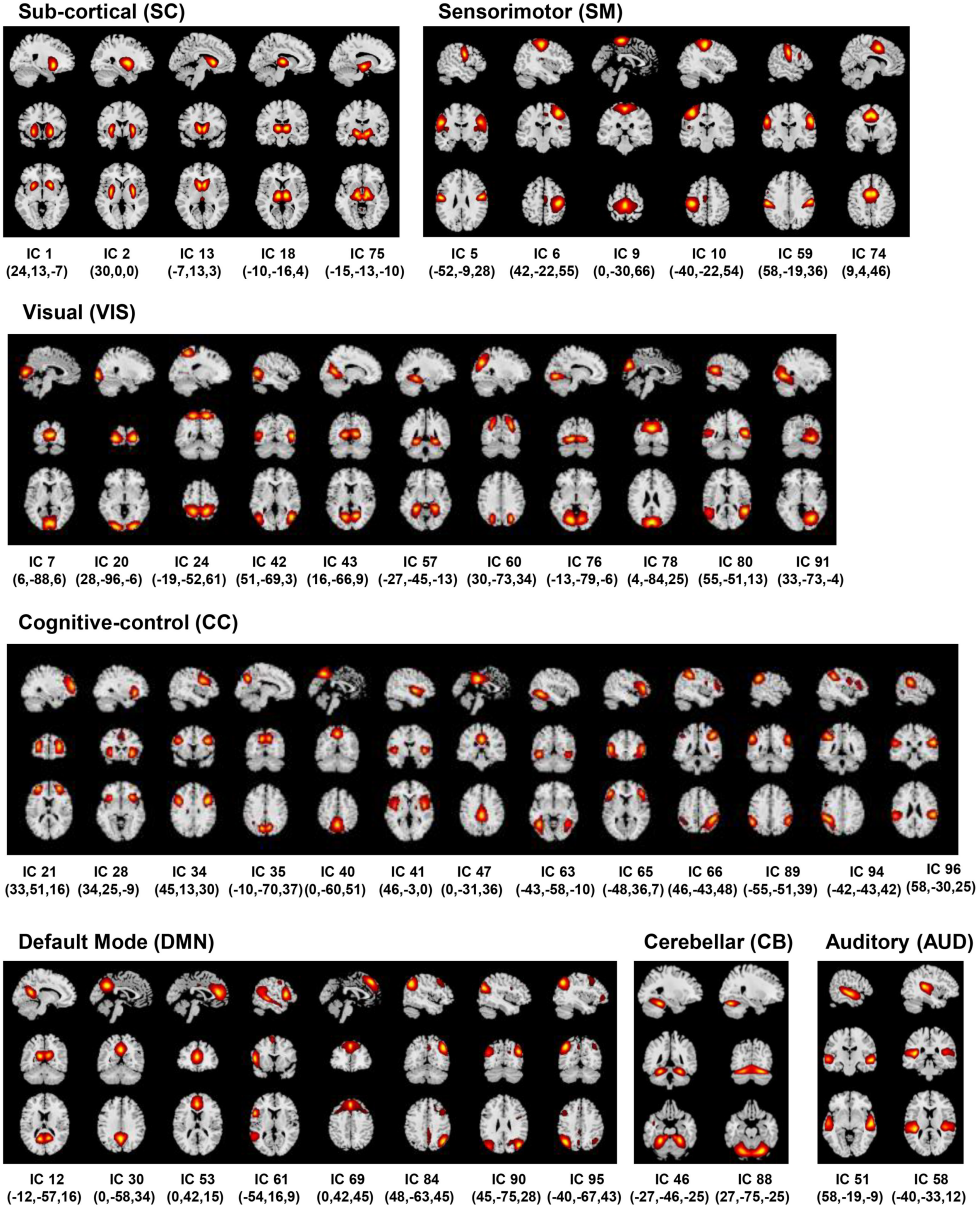
Resting State Networks (RSNs). Spatial maps of the 47 retained RSNs at the most activated sagittal, coronal and axial slices.

The back-reconstructed subject-specific SMs and TCs for the 100 independent components were extensively analyzed to identify the physiological, non-artefactual, previously established RSNs. More specifically, 47 components whose SMs showed peak activations in GM and low overlap with any known imaging, physiological, or movement-related artifacts, and mean power spectra exhibited higher low frequency content were established as RSNs for further analysis. The RSNs were assessed and distributed into the sub-cortical (SC), auditory (AUD), visual (VIS), sensorimotor (SM), attention/cognitive control (CC), default-mode (DMN), and cerebellar (CB) network domains (Figure 2).

Subject-specific TCs corresponding to the retained RSNs underwent additional post-processing steps. The TCs were de-trended to remove any existing linear, quadratic or cubic low frequency trends originating from scanner drift, orthogonalized with respect to estimated subject motion and realignment parameters, and de-spiked using AFNI's 3dDespike function to replace outlier points with values estimated from third order spline fit to neighboring portions of the TCs.

#### FC estimation and temporal variability

Similar to previous works (Allen et al., 2012; Damaraju et al., 2014), time-varying FC was estimated by sliding a window of length 22 TRs (44 s) in steps of 1 TR (2 s). This sliding window analysis used a tapered window generated by convolving a rectangular window of length 22 TRs (44 s) with a Gaussian window of standard deviation equal to 3 TRs. The window length parameter has a significant impact on the observed dynamics, however our choice of 44 s (similar to window duration as used in Damaraju et al., 2014 on the same fBIRN phase 3 dataset) falls within recommended ranges in multiple works. In background, Leonardi and Van De Ville (2015) proposed a lower limit for window length using the (inverse of minimum frequency) thumb rule, which Zalesky and Breakspear (2015) formally demonstrated to be overly conservative especially in moderate SNR conditions (i.e., relatively shorter windows than as suggested by the thumb rule can be used to capture the fluctuations in time-varying connectivity). Recent studies have reported peak maximum detection probability of time-varying fluctuations (Hindriks et al., 2016) and peaks of significance of window lengths (Liégeois et al., 2016) in a similar (40–60 s) range. Furthermore, there are several studies that corroborate that varying the window length parameter over a range beyond a certain “safety limit” did not change the overall observed dynamics (Allen et al., 2012; Li et al., 2014; Yaesoubi et al., 2015; Deng et al., 2016; Preti et al., 2016).

#### Functional data feature space estimation

The wFNC data were decomposed using temporal ICA to reveal a set of “n” aggregate connectivity patterns (or aggregate states) shared amongst subjects and a set of “n” temporally independent connectivity patterns. Notably, the “n” temporally independent connectivity patterns are a concatenation of “n” individual subject time-courses which are not independent subject-wise. We estimate the feature space for the functional data as subject-specific “versions” of the aggregate states through a modified form of spatio-temporal (dual) regression (Filippini et al., 2009; Erhardt et al., 2011). In this analysis, the aggregate states are regressed into each subject's wFNC data to obtain a set of subject-specific time-courses in the first regression step which are then regressed into each subject's wFNC data to get the subject-specific states in the second regression step. The estimated functional data feature space is next simultaneously analyzed with the GM maps estimated from structural data using the mCCA+jICA data fusion algorithm.

### The mCCA+jICA framework

As a framework to evaluate fusion of feature spaces from two imaging modalities, this method reveals flexible, i.e., both highly and weakly correlated, joint sources from both the modalities. The framework (Figure 1B) assumes the multimodal dataset (*X*_*k*_) to be a linear mixture of a (*M*) number of sources (*S*_*k*_) mixed with non-singular matrices (*A*_*k*_), where k is the modality index. Following (Sui et al., 2011, 2013), we used the minimum description length (MDL) criterion to estimate the number of independent components to be nine. Hence, we evaluate the feature spaces for a total number of nine components (*M* = 9) for both the fMRI and sMRI modalities.

In the first phase of the joint framework, the mCCA algorithm commences with dimensionality reduction of the feature spaces of each of the modalities using principal component analysis. In this work, we reduce the input data to a high (number of subjects-one) number of principal components so as to capture maximum subject level variance. Next, the canonical variates (*D*_*k*_) are estimated by maximizing the sum of squared correlations (SSQCOR) cost (Kettenring, 1971) in the “*M*” columns of canonical variates. In the final step of the first phase, the canonical correlation coefficients (CCCs) are estimated as associated maps (*C*_*k*_) by inverting the *X*_*k*_ = *D*_*k*_*C*_*k*_ model [i.e., *C*_*k*_ = *pinv(D*_*k*_)*X*_*k*_].

In the second phase of the joint framework, the estimated CCCs are concatenated [(*C*_1_..*C*_*k*_)] and input to the jICA algorithm which enables transformation of these CCCs to an orthogonal space. This decomposition reveals “*M*” maximally independent joint sources (*S*) each of which can be interpreted as a stacked form of co-varying modality-specific components i.e., *S* = *[S*_1_ …* S*_*k*_*]*. The stacked components for the different modalities share a common mixing matrix (*W*) with the jICA linear mixing model evaluated as *[C*_1_..*C*_*k*_*]* = *W [S*_1_..*S*_*k*_*]*. Hence, the effective mCCA+jICA can be summarized as *X*_*k*_ = *(D*_*k*_*W*^−*1*^*)S*_*k*_, where the effective modality-specific mixing matrices are estimated as *A*_*k*_ = *D*_*k*_*W*^−*1*^. The combined framework is illustrated in Figure 1B and further details on the parametrical/methodological choices in the algorithm can be found in the referenced original works (Sui et al., 2011, 2013).

## Results

**Figure 3:**
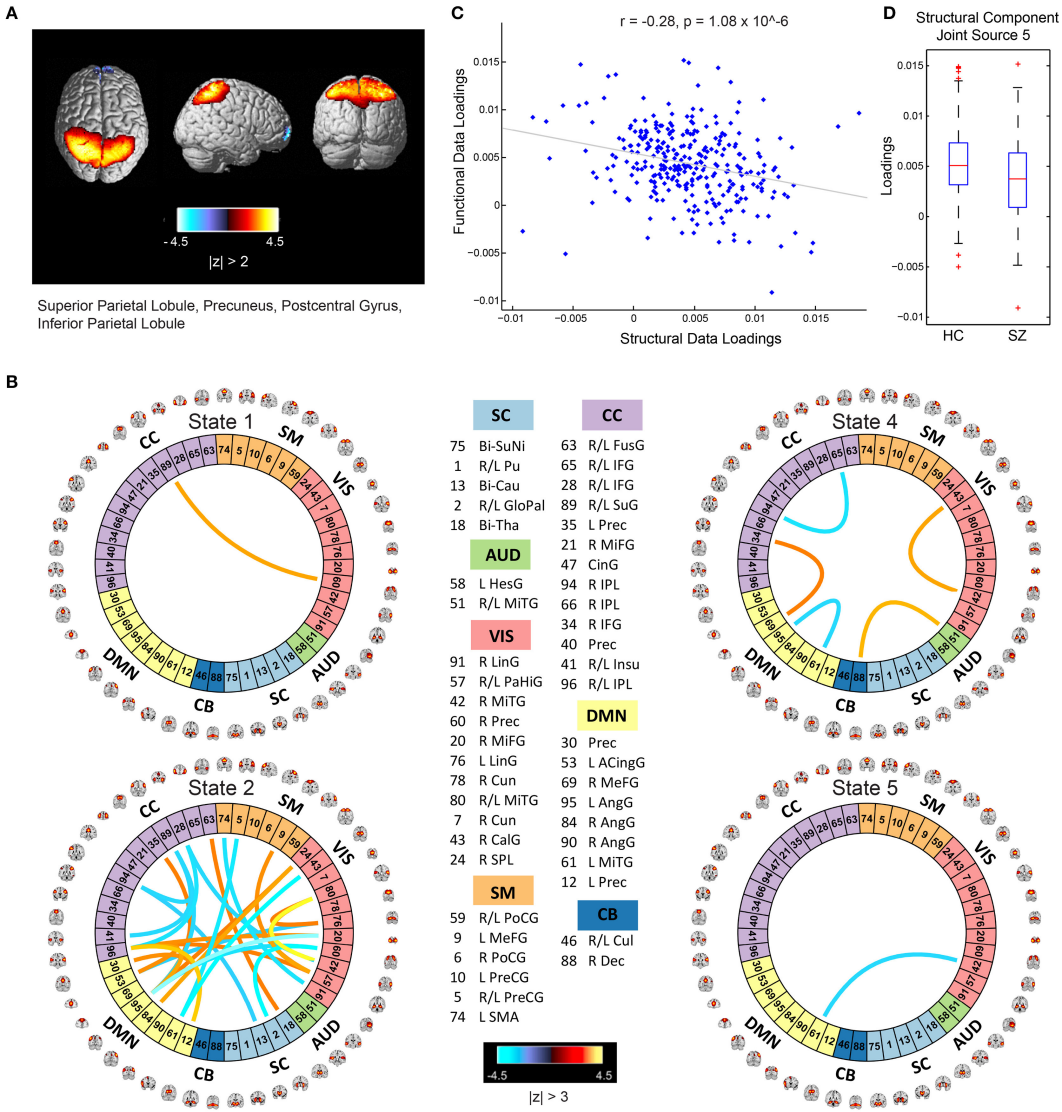
Joint Source 1. **(A)** Spatial maps of the most activated regions for the structural component in the first joint source; **(B)** A visualization of significant links (functional connections with highest connectivity strengths i.e., with *z*-scores of connectivity strengths: |*z*| > 3) and their connectivity strengths for the functional component in the first joint source; **(C)** Scatterplot of the functional data loadings with the structural data loadings revealed a significant correlation (*r* = −0.28, *p* = 1.08 × 10^−6^); and **(D)** The group mean for the loading parameters was significantly lower for participants with schizophrenia, thus suggesting significant reductions in gray matter volume for this structural component.

**Figure 4:**
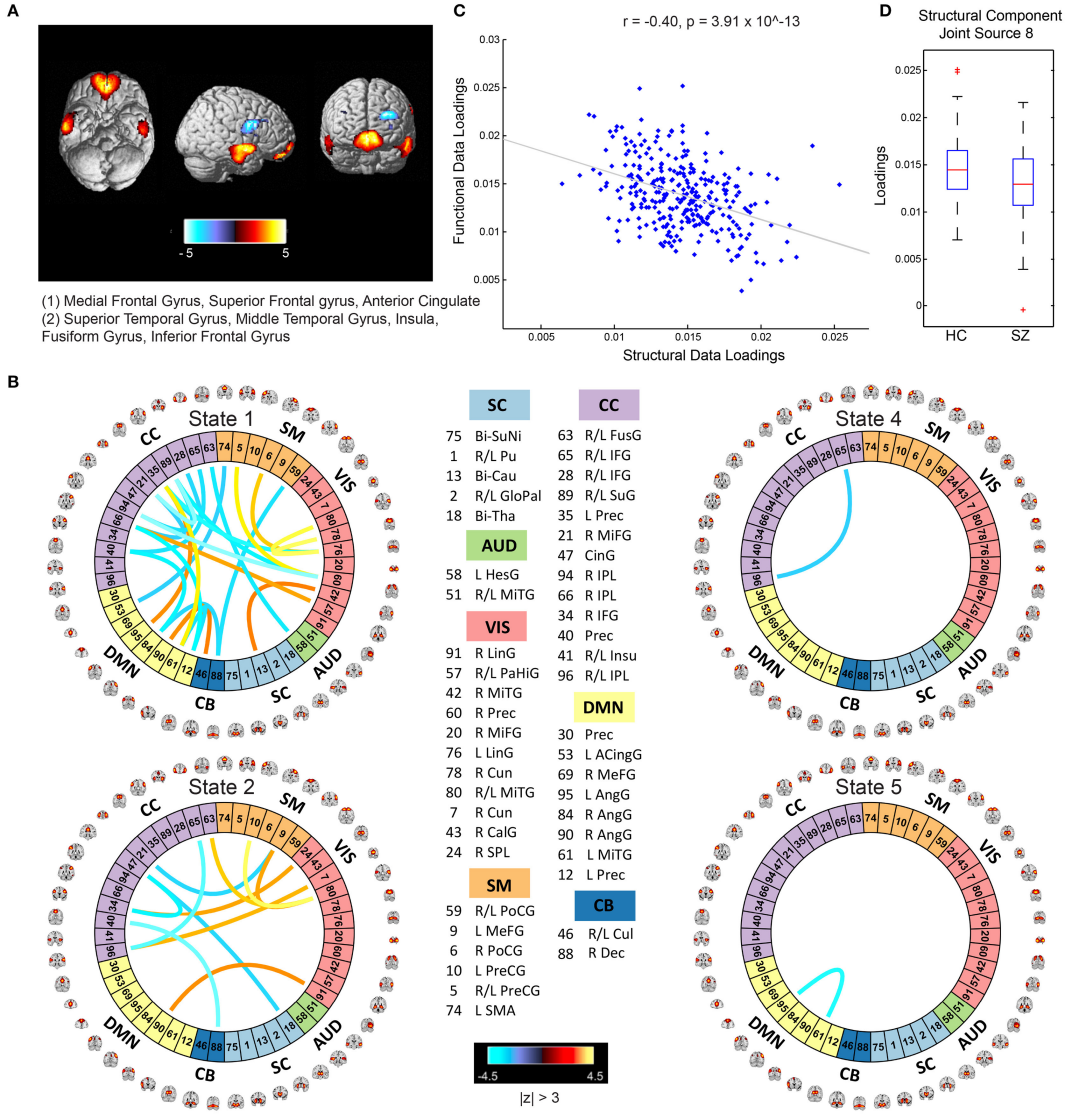
Joint Source 2. **(A)** Spatial maps of the most activated regions for the structural component in the second joint source; **(B)** A visualization of significant links (functional connections with highest connectivity strengths i.e., with *z*-scores of connectivity strengths: |*z*| > 3) and their connectivity strengths for the functional component in the second joint source; **(C)** Scatterplot of the functional data loadings with the structural data loadings revealed a significant correlation (*r* = −0.40, *p* = 3.91 × 10^−13^); and **(D)** The group mean for the loading parameters was significantly lower for participants with schizophrenia, thus suggesting significant reductions in gray matter volume for this structural component.

The mCCA+jICA framework identified two sMRI-fMRI joint sources with (1) significant correlations between their constituent structural and functional components; and (2) significant group differences in each of these constituent structural and functional components. Figures 3, 4 show the spatial maps for the constituent structural component, the connectivity strengths for the co-varying functional component's inter-regional connections and other associated results for the first and the second joint source, respectively. It must be noted that the constituent structural components in the joint sources estimated here are patterns of brain structure (i.e., clusters of brain voxels) that exhibit co-variations across subjects. These could be interpreted analogous to sources as identified with source based morphometry (SBM) (Xu et al., 2009a; Caprihan et al., 2011; Turner et al., 2012; Castro et al., 2014; Gupta et al., 2015), an approach that can be essentially considered as a multivariate extension of a voxel based approach, for example, voxel based morphometry (VBM). In both figures, for display purposes, only the high (and low) activation regions for the structural component and only the edges or connections with high (and low) connectivity strengths for the functional component are shown. More specifically, the structural component maps are lower thresholded at 25% of the maximum absolute activation value, whereas for the functional component, the inter-regional connectivity strengths, after converting to *z*-scores are thresholded at |*z*| > 3. For the functional component, we will hereon refer to the (post-thresholding) retained inter-regional connections as “significant links.”

### Joint source 1

As illustrated in Figure 3A, the structural component for the first joint source consists of peak activations in the superior parietal lobule (major constituent), precuneus, postcentral gyrus, and inferior parietal lobule. The number of significant connections in the linked functional component were high for the default mode, cognitive control and visual network domains in state 2, whereas the other states had a lot fewer total number of significant connections (five in state 4, one each in states 1 and 5, and none in state 3) as seen in Figure 3B. For this joint source, these constituent co-varying structural and functional components were found to be significantly correlated (*r* = −0.28, *p* = 1.08 × 10^−6^) as also evident from the scatterplot of their loading parameters in Figure 3C. Since negative correlation was observed, participants showing lower GM loadings generally exhibited higher connectivity strength in the functional connections. Finally, the structural component showed significant group difference (*p* = 0.0032) with a significantly lower group mean of the loadings for patients with schizophrenia (Figure 3D), whereas its linked i.e., co-varying functional correlate also showed significant group difference (*p* = 0.0072) with a significantly lower group mean for controls.

### Joint source 2

The structural component for the second joint source depicted in Figure 4A consisted of two major positively activated regions. The first major activation comprised regions from the medial frontal gyrus and superior frontal gyrus, whereas the second major activation comprised regions from the superior temporal gyrus, inferior temporal gyrus, insula, fusiform gyrus, and middle temporal gyrus. The number of significant connections in the linked functional component were high particularly for the default mode, cognitive control and visual network domains in state 1 (an observation similar to state 2 of the functional component corresponding to the first joint source) and moderate for the cognitive control, sensorimotor, and visual domains in state 2, whereas the other states had a lot fewer total number of significant connections (one each in states 4 and 5, and none in state 3) as seen in Figure 3B. For this joint source, these constituent co-varying structural and functional components were found to be significantly correlated (*r* = −0.40, *p* = 3.91 × 10^−13^). The corresponding scatterplot of their loading parameters can be seen in Figure 4C. Similar to the first joint source, since negative correlation was observed, participants showing lower GM loadings had higher connectivity strength in the functional connections. Finally, the structural component showed significant group difference (*p* = 0.0022) with a significantly reduced group mean for the schizophrenia patients, whereas its linked i.e., co-varying functional correlative also showed significant group difference (*p* = 0.0438) reflecting a significantly lower group mean for controls.

## Discussion

In this study, we investigated whether a relationship between GM and time-varying FC measures exists and if that relationship could be used to study characteristic brain aberrations in schizophrenia. Using a novel, unified framework, we first estimated distilled, i.e., (relatively) lower-dimensional feature spaces from the high-dimensional fMRI and sMRI data. Next, we performed joint analysis on the estimated feature spaces leveraging a symmetric fusion approach, mCCA+jICA, to extract jointly co-varying structural and functional components and characterize interactions between these components. In this specific section, we will discuss our results specifically addressing few important questions, such as how the co-variation in the inter-modality components could be interpreted and how the underlying associations are meaningful. We will conclude by highlighting some critical facets and limitations that could be explored in immediate future work.

Specifically, our results revealed two mCCA+jICA joint sources that featured significant correlation between their constituent modality-specific components and highlighted group differences in both of their modality-specific components. Both the joint sources showed significant negative correlations between their modality-specific constituent components (joint source 1: *r* = −0.28, *p* = 1.08 × 10^−6^; joint source 2: *r* = −0.40, *p* = 3.91 × 10^−13^), as seen in Figures 3C, 4C. This implies that from joint source 1, for a given subject, if the GM volumes in the positively activated regions in the structural component (superior parietal lobule, precuneus, postcentral gyrus and inferior parietal lobule) are estimated to be higher, it will exhibit significantly decreased connectivity strength in the inter-regional links in the functional component (i.e., the absolute magnitude of inter-regional links with positive connectivity strengths in the functional component will show significant decrease, and the absolute magnitude of inter-regional links with negative connectivity strengths in the functional component will show significant increase). Alternatively, decreased observed GM volumes in the positively activated regions would imply higher connectivity strengths in the significant functional links for that given subject. Similar inferences can be deduced for source 2 wherein changes in GM volumes in both of the positively activated distinct regions in the structural component (i.e., medial frontal gyrus and superior frontal gyrus; and superior temporal gyrus, inferior temporal gyrus, insula, fusiform gyrus, and middle temporal gyrus) would drive the estimated significant inter-regional links accordingly.

An introspection of the modality-specific components of the joint sources revealed several lines of evidence of conformance with previously reported findings in the literature as discussed next. To begin with, the structural components in both joint sources showed significant group differences in the loading parameters (joint source 1: *p* = 0.0032; joint source 2: *p* = 0.0022), with significantly lower group mean for the schizophrenia group. This suggests a significant decrease in GM volume in the brain regions depicted by these components in participants with schizophrenia. Our results are consistent with several previous studies (as discussed in detail next), where reduced GM volume in schizophrenia has been reported in the similar brain regions as identified in our structural components. The first joint source highlighted peak activations in the superior parietal lobule (major constituent), precuneus, postcentral gyrus, and inferior parietal lobule brain regions as the structural modality component (as illustrated in Figure 3A). Interestingly, a recent study on SBM and VBM evaluating GM abnormalities in schizophrenia patients also found a similar structural component showing positive activation patterns and that captured group differences between schizophrenia and healthy controls (Gupta et al., 2015). Besides, previous studies have also concluded reduced GM volume in superior parietal regions (Buchanan et al., 2004), precuneus (Hulshoff Pol et al., 2001) and postcentral gyrus (Glahn et al., 2008); thus, our work adds further evidence that abnormal patterns of GM volume in these regions play an important role as schizophrenia biomarkers. Similar evidence could be established for the significant structural component in the second joint source captured by our framework (as illustrated in Figure 4A). This structural component included two major positively activated brain regions, where one of them consisted of regions from the medial frontal gyrus, anterior cingulate and superior frontal gyrus, and the other included regions from the superior temporal gyrus, inferior temporal gyrus, insula, fusiform gyrus and middle temporal gyrus. Particularly, the anterior cingulate has been recognized as a vital structure for social cognitive processing and has been previously identified as one of the major sources of social dysfunction in schizophrenia patients (Fujiwara et al., 2007). Additionally, very-similar fronto-temporal GM changes capturing group difference between schizophrenia and healthy participants were also found in Gupta et al. (2015). In fact, there are several other studies/reviews on GM differences in schizophrenia patients that have suggested significant reduction in GM volume in the temporal and frontal cortices (Shenton et al., 2001; Thompson et al., 2001; Giuliani et al., 2005).

Significant correspondence with previously reported studies in literature could also be drawn for few evaluated significant inter-regional (i.e., inter-RSN) links in the estimated functional components. Firstly, the functional components for both of the retained joint sources showed significant connectivity links in time-varying connectivity states 1, 2, 4, and 5. For the first functional component (joint source 1), most of the significant inter-RSN links are captured in state 2, where both positive and negative connectivity strengths across various network domains can be observed (Figure 3B). In this state, RSNs in the DMN domain showed significant connectivity within themselves and with RSNs from CC, SM, and VIS domains as well. Interestingly, one of the DMN RSNs, IC95, highlighted by the brain regions in left angular gyrus, showed positive connectivity weight with a RSN from the CC domain, IC35, left precuneus. This is in line with a previous study that has shown aberrant connectivity patterns between angular gyrus and precuneus in schizophrenia patients (Rashid et al., 2014). Indeed, studies have widely reported the involvement of angular gyrus in language processing, memory and social cognition (Hall et al., 2005; Binder et al., 2009; Price, 2010; Clos et al., 2014), and abnormal connectivity patterns in schizophrenia in the precuneus, which is involved in episodic memory (Rugg and Henson, 2002), mental imagery recall (Fletcher et al., 1996), and self-processing operations (Cavanna and Trimble, 2006). Furthermore, several studies have shown strong evidence of disrupted DMN connectivity in schizophrenia patients (Garrity et al., 2007; Ongur et al., 2010), and so it would be interesting to explore significant links involving the DMN RSNs. As an example, in state 2 of this functional component (joint source 1), we observed negative connectivity strength between another DMN component (IC61: left middle temporal gyrus) and a VIS RSN (IC43: right calcarine gyrus), while the same DMN RSN (IC61) showed negative connectivity strength with a SM RSN (IC5; bi-lateral precentral gyrus) as well. For this functional component (joint source 1), we also note that the other states in this functional component (i.e., states 1, 4, and 5) showed significant inter-RSN links between VIS and CC domains (state 1), between DMN and CC, between AUD and CB domains, within DMN, CC, and VIS domains (state 4), and between DMN and VIS domains (state 5). Furthermore, an examination of the functional component from joint source 2 revealed some interesting significant links in state 1, the most densely connected state (Figure 4B). In this state, a DMN RSN (IC95; left angular gyrus) showed significant positive connectivity strength with a CC RSN (IC35; left precuneus), an observation also found in state 2 emergent in the first joint source. Again, similar to state 2 from the first joint source, another DMN RSN (IC61; left middle temporal gyrus) showed significantly positive connectivity strength with the same CC RSN (IC35; left precuneus). In fact, dysfunctional temporal lobe connectivity has been reported in several schizophrenia connectivity studies (Shenton et al., 1992; Ford et al., 2002), suggesting that networks from the temporal regions play a significant role in schizophrenia etiology. Finally, for this functional component (joint source 2) the other states (i.e., states 2, 4 and 5) showed significant inter-RSN links from the DMN, CC and SM and CB domains.

While we closely evaluate a few interesting connections in scope of this work, there is much more that could be done to evaluate these results to further enhance our understanding of the structure-function relationships and further contribute to characterizing schizophrenia. In the specific context of findings from our mCCA+jICA based framework as studied in this paper, it would be most appropriate to first extensively validate the significant findings in a future analysis evaluating multiple multimodal datasets featuring schizophrenia participants. We also note that while different combinations of cost functions and model orders can yield similar results, they can also introduce decompositions different to a degree; hence, comparing performance of the mCCA+jICA approach for a range of these parameters would be another interesting future work. Further investigations could also benefit from evaluating associations of sleep indices and schizophrenia risk factors with the structural and functional component patterns. We anticipate that similar methods could be easily extended to the study of other brain conditions; likewise, different feature spaces, combinations of neuroimaging modalities and algorithms could be evaluated in the proposed fashion.

## Conclusion

Multimodal data fusion through symmetric approaches provides an opportunity to understand brain complexities. Using a multivariate symmetric fusion approach, we were able to identify co-varying GM and time-varying FC components that revealed disrupted links in schizophrenia. We suggest that studying such interactions can provide a useful way of evaluating structure-function relationships and characterizing schizophrenia or other brain conditions.

## Ethics statement

This study used data collected in accordance with the local institutional review boards at the University of California Irvine (UCI), Los Angeles (UCLA); University of New Mexico (NM), University of Iowa (IA), University of Minnesota (MN), Duke University/University of North Carolina, University of California San Diego (UCSD; healthy subjects only), and the University of California San Francisco (UCSF). All subjects gave written informed consent in accordance with the Declaration of Helsinki before participation. The study protocol was approved by the institutional review board at the University of New Mexico. All studies were collected in accordance with the local (site-specific) institutional review board guidelines with written informed consent from all subjects.

## Author contributions

AA and VC designed the research. AA, SR, and ED performed the research. AA, BR, and VC analyzed the results. AA, BR, and VC wrote the paper.

### Conflict of interest statement

The authors declare that the research was conducted in the absence of any commercial or financial relationships that could be construed as a potential conflict of interest.
